# AI in Medicine, Covid-19 and Springer Nature's Open Access Agreement

**DOI:** 10.1007/s13218-020-00661-y

**Published:** 2020-06-03

**Authors:** Daniel Sonntag

**Affiliations:** German Research Center for Artificial Intelligence (DFKI), Saarbrücken, Germany

Dear readers,

Let’s say something about applications of AI in Medicine first. They include intelligent interactive monitoring of patient’s environment and needs, intelligent interfaces supporting access to healthcare services, and patient-tailored decision support. Just to name a few. In addition, they include decision support systems especially for the doctor, which model the diagnostic reasoning and decision-making of medical experts. In recent years, prominent examples also include machine learning in medical imaging; deep learning for identifying metastatic breast cancer on images seems promising. Or in non-imaging domains, massively multitask networks for drug discovery.

This means, AI is interdisciplinary in medicine, a real general purpose technology around images, texts, and biodata. At least four application fields become an ally and seem promising in the fight against Covid-19. (Although AI has not yet made an impact.) The community, you, have taken up the challenge. First, medical imaging might help to spot Covid-19 in x-rays. This recent development might however never result into a production-ready solution, Berlin Charité’s lab solution with monovettes just seem more appropriate. The second application field is gene sequencing. Commercial companies such as DeepMind offer data and contribute to the scientific effort by releasing structure predictions of several understudied proteins associated with Covid-19. The third application field is language technology and text mining. The Allen institute for AI creates a Kaggle challenge, issuing a call to action to AI experts to develop text mining solutions. The Covid-19 Open Research (CORD-19) dataset represents an extensive machine-readable coronavirus literature collection. The fourth application field is disaster management. Only recently, BMBF announces a call about national disaster management, focusing on natural disasters such as flood. Covid-19 management would be welcome.

Coming to the two main points, to use AI methods to a larger extent, we need a systematic collection of patient information and medical literature in a digital format, and machine learning solutions for end users. Digital records can be shared across different healthcare settings, store data accurately, and capture the state of a patient across time. In addition, open source software and open access scientific papers and books around AI and medicine have to be made available.

Just at the right time, we are happy to announce Springer Nature's Open Access Agreement (read more in the News section of the KI Journal): if you are a corresponding author affiliated with a German university or research institution, you are entitled to publish open access in our KI Journal with fees covered by the German DEAL agreement. This means everyone in the German AI research community can, from now on, publish open access in our KI Journal for free, with Scopus Index!

Now to the current special issue on Interactive Machine Learning, which could be defined as the design and implementation of algorithms and intelligent user interface frameworks that facilitate machine learning with the help of human interaction. With the convergence of artificial intelligence and machine learning, and the aforementioned need of machine learning tools for medical end users (cf. the ongoing coronavirus pandemic), I am happy we have such a strong topic, i.e., challenges in interactive machine learning (and explainable AI), in this special issue. My thanks go out to the guest editors Stefano Teso and Oliver Hinz, the liaison editor Kristian Kersting, the reviewers of these papers, and Springer Nature.

Stay healthy,

Daniel Sonntag
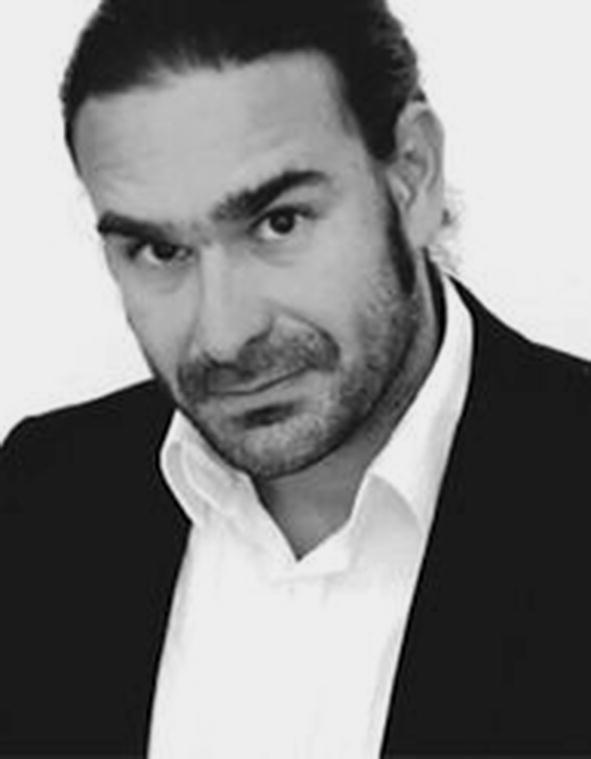


## Forthcoming Special Issues

### Two Special Issues on Ontologies and Data Management (1 and 2)

Guest editors: Thomas Schneider (University of Bremen, Germany) and Mantas Simkus (TU Wien, Austria). Liaison editor: Anni-Yasmin Turhan (Dresden University of Technology).

This special issue focuses on the theory and practice of applying ontologies in data management (ODM), which is a research topic of significant interest in Knowledge Representation and Reasoning (KR&R) and Database Theory. Modern data-centric software systems need to handle data that is often heterogeneous, sensitive, very large, and even incomplete or inconsistent. Moreover, it often has very complex structures. Thus, the development of proper tools and techniques to handle this complexity is a pressing task. Ontologies in combination with automated reasoning are acknowledged as a promising tool to address some of these challenges, and thus are receiving significant attention both among researchers and industry. For instance, the prominent data integration paradigm called ontology-based data access (OBDA) suggests the use of ontologies to provide a conceptual view of a problem domain, where various possibly heterogeneous data sources can be linked to the same ontology using mappings, enabling users to pose queries using the ontology vocabulary. For query answering in OBDA, automated reasoning is used to compile information from the sources, possibly employing the domain knowledge in the ontology to infer new information.

The special issue welcomes contributions on all aspects of ontologies and data management, including but not limited to:

Query answering: standard semantics, bag semantics, inconsistency-tolerant semantics.

Further inference tasks in the presence of data: learning, materialization, non-monotonic reasoning.

Decidability and complexity analyses.

Ontology languages and extensions: description logics (DLs), rule-based languages, first-order logic.

Combinations of ontology languages with other formalisms such as temporal logic, probabilities, action formalisms.

Applications related to ODM.

Systems and tools related to ODM.

If you are interested in contributing to this special issue, please contact the guest editors. The call is announced on the KI Journal website.

## Special Issue on Developmental Robotics

Guest editors: Manfred Eppe (University of Hamburg), Verena V. Hafner (HU Berlin), Yukie Nagai (University of Tokyo), Stefan Wermter (University of Hamburg). Liaison editor: Britta Wrede (Bielefeld University).

Human intelligence develops through experience, robot intelligence is engineered—is it? At least in the mainstream approaches based on classical Artificial Intelligence (AI) and Machine Learning (ML) the robotic engineering approach is pursued and data- or knowledge-based algorithms are designed to improve a robot’s problem-solving performance. Based on this engineering perspective of classical AI/ML approaches plenty of valuable application-specific impact has been achieved. Yet, the achievements are often subject to restrictions that involve domain knowledge as well as constraints concerning application domains and computational hardware.

Developmental Robotics seeks to extend this constrained perspective of engineered artificial robotic cognition, by building on inspiration from biological developmental processes to design robots that learn in an open-ended continuous fashion. Developmental Robotics considers cognitive domains that involve problem-solving, self-perception, developmental disorders and embodied cognition. This perspective helps to improve the performance of intelligent robotic agents, and it has already led to significant contributions that inspired cutting-edge application-oriented Machine Learning technology. In addition, Developmental Robotics also provides functional computational models that help to understand and to investigate embodied cognitive processes. For this special issue, we welcome contributions that include, but are not limited to the following topics:

Robotic self-perception and body representation; Typical development and developmental disorders; Neural foundations of development and learning; Continual learning; Transfer learning; Embodied cognition; Problem-solving; Predictive models; Intrinsic motivation; Language learning.

The KI Journal, published and indexed by Springer, supports a variety of formats including technical articles, project descriptions, survey articles, discussions, dissertation abstracts, conference reports, and book reviews. Interested authors are encouraged to contact the guest editors at their earliest convenience (also see our KI Journal website).

## Special Issue on Education in Artificial Intelligence K-12

Guest editors: Gerald Steinbauer (Graz University of Technology), Martin Kandlhofer (Austrian Computer Society), Tara Chklovski (Technovation USA), Fredrik Heintz (Linköping University) Sweden, Sven Koenig (University of Southern California). Liaison editor: Ubbo Visser (University of Miami).

The upcoming special issue of the KI Magazin addresses the emerging topic of education in Artificial Intelligence (AI) at the K-12 level. In recent years, Artificial Intelligence (AI) has attracted a lot of attention from the public, and become a major topic of economic and societal discussion. AI already has a significant influence on various areas of life and across different sectors and fields. The speed and force with which AI is impacting our work and everyday life poses a tremendous challenge for our society and educational system. Teaching fundamental AI concepts and techniques has traditionally been done at the university level. However, in recent years several initiatives and projects pursuing the mission of K-12 AI education have emerged. In this context we also see education organizations and AI experts as well as governments developing and deploying AI-curricula and programs for a K-12 audience. The aim of this special issue is to provide a compact overview over this growing field. We invite contributions from researchers, practitioners, and educators interested in education in AI at K-12 level. If you are interested in submitting a paper, please contact one of the guest editors.

